# Herpes Virus Infection Is Associated with Vascular Remodeling and Pulmonary Hypertension in Idiopathic Pulmonary Fibrosis

**DOI:** 10.1371/journal.pone.0055715

**Published:** 2013-02-28

**Authors:** Fiorella Calabrese, Anja Kipar, Francesca Lunardi, Elisabetta Balestro, Egle Perissinotto, Emanuela Rossi, Nazarena Nannini, Giuseppe Marulli, James P. Stewart, Federico Rea

**Affiliations:** 1 Department of Cardiac, Thoracic and Vascular Sciences, University of Padova, Padova, Italy; 2 Department of Infection Biology, University of Liverpool, Liverpool, United Kingdom; 3 School of Veterinary Science, University of Liverpool, Liverpool, United Kingdom; 4 Veterinary Pathology, Department of Basic Veterinary Science, Faculty of Veterinary Medicine, University of Helsinki, Helsinki, Finland; McMaster University, Canada

## Abstract

**Background:**

Pulmonary hypertension (PH) represents an important complication of idiopathic pulmonary fibrosis (IPF) with a negative impact on patient survival. Herpes viruses are thought to play an etiological role in the development and/or progression of IPF. The influence of viruses on PH associated with IPF is unknown. We aimed to investigate the influence of viruses in IPF patients focusing on aspects related to PH. A laboratory mouse model of gamma-herpesvirus (MHV-68) induced pulmonary fibrosis was also assessed.

**Methods:**

Lung tissue samples from 55 IPF patients and 41 controls were studied by molecular analysis to detect various viral genomes. Viral molecular data obtained were correlated with mean pulmonary arterial pressure (mPAP) and arterial remodelling. Different clinical and morphological variables were studied by univariate and multivariate analyses at time of transplant and in the early post-transplant period. The same lung tissue analyses were performed in MHV-68 infected mice.

**Results:**

A higher frequency of virus positive cases was found in IPF patients than in controls (*p = *0.0003) and only herpes virus genomes were detected. Viral cases showed higher mPAP (*p = *0.01), poorer performance in the six minute walking test (6MWT; *p = *0.002) and higher frequency of primary graft (PGD) dysfunction after lung transplant (p = 0.02). Increased arterial thickening, particularly of the intimal layer (*p = *0.002 and *p = *0.004) and higher TGF-β expression (*p = *0.002) were demonstrated in viral cases. The remodelled vessels showed increased vessel cell proliferation (Ki-67 positive cells) in the proximity to metaplastic epithelial cells and macrophages. Viral infection was associated with higher mPAP (*p = *0.03), poorer performance in the 6MWT (*p = *0.008) and PGD (*p* = 0.02) after adjusting for other covariates/intermediate factors. In MHV-68 infected mice, morphological features were similar to those of patients.

**Conclusion:**

Herpesviral infections may contribute to the development of PH in IPF patients.

## Introduction

Idiopathic pulmonary fibrosis (IPF), morphologically usual interstitial pneumonia, is a chronic progressive disease of unknown aetiology with irreversible scarring in the lung [1]. No proven effective treatment is available other than lung transplantation. Survival time is estimated at 2.5 years after initial diagnosis, although disease progression is highly variable [2], [3]. Acute exacerbation, lung cancer and pulmonary hypertension (PH) are the main complications which adversely affect the survival of IPF patients. Different from acute exacerbation which can occur at any stage of the disease, lung cancer and pulmonary hypertension (PH) are more likely in advanced disease. For the latter, a frequency of 20–90% has been reported and it carries severe consequences including decreased exercise capacity and increased mortality before and after lung transplantation [4]–[7].

Recent studies have shown that PH measured with right-heart catheterization or transthoracic echocardiography in IPF patients is associated with low DLCO, shorter walk distances, and desaturation during exercise [5], [6]. Similar relationships have been identified when brain natriuretic peptide was used as a surrogate marker for PH in IPF patients [8].

However to date no clinical or biohumoral parameters are available as predictive markers of PH in IPF patients. Several authors have shown that PH in IPF relates poorly to the degree of pulmonary function test and is not always confined to advanced disease [6]. Indeed even in end stage IPF cohorts, no association between the presence of PH and severity of the disease (both in terms of functional parameters and fibrosis extension) has been observed [6], [9].

Pathogenic mechanisms of IPF and of its main complications are complex and still largely unknown although different hypotheses have been proposed, varying from previous chronic inflammation with subsequent widespread fibrosis to abnormal wound healing. Increasing evidence suggests that a key event in IPF is deregulated epithelial cell function with ongoing alveolar epithelial injury and an associated abnormal host repair response, leading to patchy and heterogeneous morphological changes [10]. The complex biological processes that underlie pulmonary fibrosis might directly contribute to the pathogenesis of vascular remodelling which is equally patchy and heterogeneous. Fibrotic areas have fewer blood vessels but adjacent, non fibrotic tissues seem highly vascularised [11]. Structural changes range from capillary loss to complex vascular lesions with intima, media thickening and adventitial fibrosis. Endothelial injury, usually occurring through apoptotic cell death is another crucial aspect in the pathogenesis of IPF-associated PH as reported in recent studies [12]–[14]. Different types of mediators, including those released by injured endothelial cells, may be involved in vascular remodelling by influencing vessel smooth muscle or adventitial cell proliferation. Several studies have implicated viral infection as a cause of epithelial injury and therefore an important factor in its pathogenesis [15]–[34]. Among viral agents herpes viruses, in particular Epstein Barr virus (EBV), have been suggested as principal cofactors (as initiating or exacerbating agents) of fibrotic lung disease and treatment with ganciclovir has been shown to attenuate disease progression in a subgroup of patients [35].

Alveolar epithelial cells, one of the most important targets of the virus, are then involved in the activation of different signalling pathways responsible for fibrotic lung remodelling. In viral animal and cell models several profibrogenic cytokines/chemokines have been shown to be over-expressed and among these transforming growth factor beta 1 (TGF-β1) plays a key role [27], [32]–[34]. While many works, experimental and clinical, have highlighted the influence of EBV in promoting fibrotic parenchymal remodelling, its influence on endothelial injury and vessel remodelling and other related parameters of PH has not been yet assessed.

The aim of the study was to investigate the relevance of respiratory viruses in IPF patients, with special emphasis on cases complicated by PH. For this purpose a large number of IPF patients and control subjects including other non-IPF diffuse parenchymal lung diseases (DPLDs) were studied. Viral molecular data were correlated with mean pulmonary arterial pressure (mPAP) and arterial remodelling. Different clinical and morphological variables were studied by univariate and multivariate analysis at time of transplant and in the early post-transplant period.

A mouse model was used to examine whether murine gammaherpesvirus-68 (MHV-68), genetically and biologically similar to EBV, contributes to the development of vessel remodelling other than pulmonary fibrosis.

## Materials and Methods

### Study Population

Native lungs from 55 IPF patients who underwent lung transplantation (LT) between September 1998 and February 2010 in our Centre were studied (39 males and 16 females; mean age: 55.2±9.2 yrs, 33 single vs 22 bilateral LT). The control group consisted of 22 native lungs from other non-IPF -DPLDs- (8 males and 14 females; mean age: 44±11.4 yrs, 11 lymphangioleiomyomatosis, 4 Langerhans-cell histiocytosis, 2 sarcoidosis, 2 hypersensitivity pneumonitis, 1 non-specific interstitial pneumonia, 1 desquamative interstitial pneumonia, 1 scleroderma lung fibrosis) and 19 normal lungs (13 non implanted donor lungs and 6 autopsy cases). Prior to LT after the diagnosis of IPF, the majority received oral prednisone while a minority were treated with prednisone and azathioprine.

Each patient underwent pulmonary function testing, high resolution computed tomography and right heart catheterization. These tests were performed in all patients at the time of waiting list inclusion and before LT. For the present study clinical/functional parameters collected at the time of LT were considered.

The diagnosis of interstitial lung disease was based on the diagnostic criteria of the American Thoracic Society/European Respiratory Society Consensus Classification System [1]. The clinical history was collected, with special emphasis on age at onset of symptoms, smoking history, occupational exposure, co-morbidities and therapy (medical and non medical).

Moreover, short term post-transplant follow-up (12 months after LT) was also considered in all IPF cases, mainly focusing on primary graft dysfunction (PGD) and acute rejection, both considered causes of early and late graft dysfunction. PGD and acute rejection were graded as proposed by the International Society for Heart and Lung Transplantation (ISHLT) Working Group [36], [37]. Acute rejection was evaluated in 143 scheduled transbronchial biopsies collected in the first post-transplant year. These data have been presented at the 2010 ISHLT annual meeting.

Written informed consent was obtained from all subjects. The work was approved by the Institutional Ethics Committee. Relevant clinical data of the IPF and non-IPF DPLD population are summarized in Table 1.

**Table 1:** Study subjects (IPF patients and non-IPF DPLDs).

IPF patients (n = 55)		DPLDs (n = 22)	
Age at transplantation (years)	55.1 (7.9)	44.1 (12.6)	
Type of transplantation (bilateral)	22 (40.0%)	16 (72.7%)	
Men	39 (70.9%)	9 (40.2%)	
BMI	27.1 (4.0)	23.4 (5.2)	
Smoking status			
never smoked	9 (16.4%)	10 (45.4%)	
previous smoker	46 (83.6%)	7 (31.8%)	
History of environmental exposure	3 (5.5%)	0	
Co-morbidities			
Gastritis	5 (9.1%)	1 (4.5%)	
Diabetes	6 (10.9%)	0	
CHD	4 (7.3%)	0	
Obesity	13 (23.6%)	2 (9.1%)	
FEV1 (% of predicted value)	47.9 (16.2)	39.2 (25.3)	
FVC (% of predicted value)	45.6 (14.5)	49.0 (22.4)	
VC (% of predicted value)	43.4 (14.3)	51.0 (21.0)	
TLC (% of predicted value)	53.4 (15.4)	71.4 (20.9)	
DLco (% of predicted value)	26.0 (17.0)	23.5 (20.5)	
mPAP	24.0 (8.9)	28.8 (15.4)	

Data are number of patients (%) or mean (sd). IPF: idiopathic pulmonary fibrosis;

DPLDs: Diffuse parenchymal lung diseases; BMI: body mass index; CHD: coronary heart disease.

### Molecular Viral Detection

Nucleic acids were extracted from fresh lung tissues sampled from different areas (∼1 mg of tissue) of all 96 cases (55 IPF patients and 41 control subjects) using a modified RNAzol method [38]. Reverse transcriptase (RT)-polymerase chain reaction (PCR), PCR or nested-PCR were used to detect principal respiratory viral genomes: adenovirus, cytomegalovirus (CMV), EBV, rhinovirus, influenzavirus A and B, metapneumovirus, herpes virus (HHV)-6, HHV-7, HHV-8, parvovirus (PV) B19, parainfluenzavirus 1 and 3 and respiratory syncytial virus (as heminested PCR) as previously reported [39]. The oligonucleotides used to ascertain the quality of extracted RNA or DNA were complementary to the mRNA glyceraldehyde-3-phosphate dehydrogenize (3GPDH) and β-globin gene, respectively [40], [41,]. All samples were processed alongside negative controls (i.e. reaction mixture without DNA or RNA template) and positive (virally infected cells). Precautions were taken to avoid false positive results due to contamination by PCR product carry over, by strictly following guidelines for the general handling of the PCR procedure, such as separation of rooms, boards, and lab benches [42]. Sensitivity of PCR in our laboratory has beforehand been reported [43].

Samples were considered as true positive when the reproducibility of PCR analysis was verified a second time. Amplicon specificity was verified by direct cycle sequencing as previously described [39].

Since EBV was identified with high frequency in IPF patients, RNA-in situ hybridization-ISH (Epstein-Barr encoded RNAs, Biogenex, San Ramon, CA) was performed to identify the viral target cells. To quantify DNA viral genome copies real time PCR was also carried out in EBV positive cases whose additional DNA was available using artus® EBV TM PCR KIT [44].

### Analysis of Fibrosis Extension, TGF-β Expression and Vessel Remodelling

The extent of fibrosis was evaluated in Masson’s trichrome stained sections analyzing 10 random fields using computer-assisted morphometric software (Image ProPlus® 5.1), as previously reported [45]. The immunohistochemical detection and quantification of the profibrotic cytokine TGF-β (mouse monoclonal anti-TGF-β NovoCastra, Newcastle, UK), was performed as described before: immunostaining scores were based on the products of percentage positive cells multiplied by stain intensity (0 = negative, 1 = weak, 2 = moderate, 3 = strong) in three different high power fields. Control sections were stained without the primary antibody, without primary and secondary antibodies, or with normal sera to control for background reactivity [45].

Arterial thickening was quantified using computer-assisted morphometric software on elastic-Van Gieson (EVG) stained lung sections focusing on muscular arteries of an average diameter of 300 µm (range: 100–500 µm). In particular, medial thickening (MT) was evaluated as previously described in at least 5 arterial sections. The same approach was used to measure intimal thickening (IT): MT% = (2 × medial layer thickening/external diameter) × 100; IT% = (2 × intimal layer thickening/external diameter) × 100 [46]. The assessment of vascular remodelling also included an evaluation of apoptosis and proliferation of vessel cell components using the TUNEL technique and Ki-67 immunostaining respectively, as previously described [47]. Briefly, for TUNEL, after proteinase K (Boehringer, Mannheim, Germany) digestion at a concentration of 20 µg/ml, the slides were incubated with TdT/biotinylated dUTP diluted in buffer (Boehringer, Mannheim, Germany) and any labeling visualized with 3-3′-diaminobenzidine and 30 ml hydrogen peroxide. Slides incubated in buffer without TdT or biotinylated UTP served as negative controls and slides incubated with 1 µg/ml DNAse (Sigma-Aldrich, Milan, Italy) served as positive controls.

For Ki-67 immunohistochemistry, after microwave antigen retrieval, the sections were treated with normal serum (Immunotech, Marseille, France) and incubated for 60 min with the primary monoclonal antibody anti-Ki67 (MIB-1, Gene Tex, Irvine, CA) at a dilution of 1∶100. Sections were subsequently incubated with rabbit HRP polymer (Dako, Glostrup, Denmark) for 30 min. Immunoreactivity was visualized with 3-3′-diaminobenzidine (Dako). Negative controls processed omitting the primary antibody did not show any reaction. Ki-67 expression and TUNEL positivity were evaluated only in blood vessel cell components. All evaluations were performed blindly.

### Animal Model

Female, 5 to 8 week old CD-1 mice [Hsd:ICR (CD1)] were purchased from Harlan Laboratories, UK and housed at the University of Liverpool under specific pathogen-free conditions. Mice were intranasally infected with 4×10^5^ PFU MHV-68 and euthanized 7 days (n = 3), 14 days (n = 6) and 23 days (n = 3) post infection (post infection). Uninfected mice (n = 4) served as controls.

Immediately after death, lungs were collected and parts frozen at −80°C for RNA extraction: others were fixed in 10% buffered formalin and routinely embedded in paraffin wax. All experiments were performed in strict accordance with the recommendations in the Guide for the Care and Use of Laboratory Animals of The National Institutes of Health. The protocol was approved by UK Home Office regulations and under Project Licence number 40/2483 and Personal Licence number 60/6501. All infections were performed under light isoflorane anaesthesia. All experiments were carried out in manner to minimize suffering.

### Analysis of Fibrosis Extension, TGF-β Expression and Vessel Remodelling

Consecutive paraffin-embedded sections were stained with haematoxylin and eosin for histology, with Masson’s trichrome stain for fibrosis extension and with EVG for vessel remodelling, both morphometrically measured as above. The evaluation of arterial thickening focused on pulmonary arteries with an average diameter of 82 µm ranging from 43 to 100 µm. The assessment of vascular remodelling also included an evaluation of apoptosis and proliferation of vessel cell components using the TUNEL technique and Ki-67 immunostaining, as described above. Ki-67 antibody used for this analysis was rabbit polyclonal (Abcam, Cambridge, UK). Immunohistochemistry was also employed for the detection of MHV-68 antigen, to highlight lung macrophages (expression of lysozyme) and to demonstrate TGF-β expression (Genetex, Irvane, CA). Viral tRNA was demonstrated by RNA-ISH [48].

### Statistical Analysis

Data were analyzed using the SAS statistical software version 9.1.3 (SAS Institute, Cary, NC, USA). For quantitative variables the results are expressed as mean values ± standard deviation if normally distributed, otherwise as median, Q1–Q3. The normality of distribution of quantitative variables was tested by means of Shapiro-Wilk statistics. For quantitative characteristics, differences between subjects with and without viral infection were evaluated by using the Mann-Whitney test. The prevalence of specific conditions was expressed as a percentage, and differences between groups were evaluated using the χ-squared test and exact Fisher’s test, as adequate.

Unadjusted and adjusted relationships between virus and clinical/morphological features were tested with general linear models (GLM procedure). In all analyses, a two-tail level for significance was set at 0.05. Simple logistic regression models were applied to estimate odds ratios (ORs) for PGD using recipients’ and donors’ characteristics as predictors. A multivariate logistic model was used to estimate the adjusted OR of virus for PGD, controlling for PAP>25 mmHg. Considering that incidence was quite common in the study population, adjusted risk ratios (RRs) were approximated from adjusted ORs [49].

## Results

### Study Population

#### Viral data, tissue and vessel remodelling

**Figure 1:**
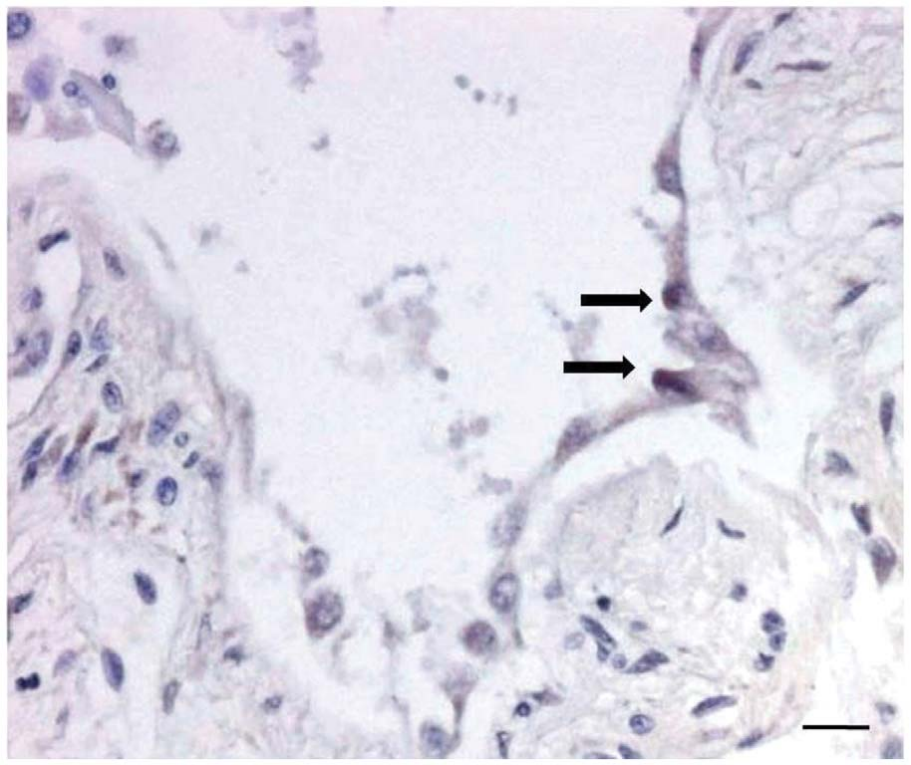
In situ hybridization for EBV. EBER transcripts well seen in the nuclei of two alveolar epithelial cells (arrows). Bar scale: 10 µm.

Patients with IPF showed a higher frequency of viral infection than control cases (40% vs 7.3%; *p = *0.0003). Normal lungs were all negative. Herpes viruses were the only detected genomes in IPF and EBV resulted as the most frequent, present in more than half of IPF patients. In 2 cases double infection was found. EBV was never detected in control cases (DPLDs and normal lungs). Gene sequencing of all amplicons showed a high homology (from 95% to 99%) with human viral genome sequences. RNA-ISH for EBV (EBER) identified viral RNA in 40% of EBV-PCR positive IPF cases. The positivity was detected in alveolar epithelial cells other than within monocytes/macrophages (Figure 1). Real time PCR showed a high number of EBV genome copies (mean±SD:1085000±120208 copies/µl DNA). The extent of fibrosis in IPF lungs showed a mean of 36.7±12.3% (range: 14.6%–65.3%) and was significantly higher than in the DPLD control group (36.7±12.3% vs 15.4±15.4%, p<0.0001). Normal lungs from donors showed no evidence of pathological remodelling.

TGF-β expression in IPF lungs was mainly detected in macrophages (median, Q1–Q3: 100, 20–210) and metaplastic alveolar epithelial cells (120, 70–210). Median TGF-β scores in the alveolar epithelium were significantly higher in IPF patients than in the DPLD group (120, 70–210 vs 0, 0–0, p<0.0001). In normal lungs, TGF-β expression was restricted to only scattered intraalveolar macrophages. Arterial remodelling in IPF cases showed a median total thickness score of 43.8% (Q1–Q3: 35.7–53.1%) with IT and MT scores of 17.7% (Q1–Q3: 13.5–24%) and 26.2% (Q1–Q3: 21.8–28.9%) respectively. These values were significantly lower in the DPLD group (total thickness: 37.9% vs 43.8%, *p = *0.03; IT: 12.3% vs 17.7%, *p = *0.005). Normal lungs did not show arterial remodelling (Table 2).

**Table 2:** Viral genome frequency and pathological data in IPF and control cases.

	IPF patients (55)	Control cases (41)*	*p-values*
**Virus positive cases**	22/55 (40%)	3/41 (7.3%)	*0.0003*
EBV	13/22 (59.1%)	0	
HHV-6	7/22 (31.8%)	1/3 (33.3%)**	
CMV	4/22 (18.2%)	2/3 (66.7%)**	
PVB19	0	1/3 (33.3%)**	
Fibrotic extension, mean % (SD)	36.7 (12.3)	15.4 (15.4)***	*<0.0001*
Medial arterial remodelling, median % (Q1–Q3)	26.2 (21.8–28.9)	22.4 (18.9–27.5)***	*ns*
Intimal arterial remodelling, median % (Q1–Q3)	17.7 (13.5–24.0)	12.3 (9.0–17.0) ***	*0.005*
Total arterial remodelling, median % (Q1–Q3)	43.8 (35.7–53.1)	37.9 (31.7–44.6) ***	*0.03*
Macrophagic TGF-ß score, median % (Q1–Q3)	100 (20–210)	50 (10–180) ***	*ns*
Alveolar epithelial TGF-β score, median % (Q1–Q3)	120 (70–210)	0 (0–0)***	*<0.0001*

IPF: idiopathic pulmonary fibrosis; EBV: Epstein Barr virus; HHV6: herpes virus 6; CMV: cytomegalovirus PV: parvovirus; TGF : transforming growth factor.

* All donor lungs were negative;

** these viruses were detected only in patients with LAM.

Double infections were detected in two IPF patients and 1 control case.

*** These parameters were quantified only in DPLD patients, not in normal lungs.

#### Correlation between viral molecular data and clinical/morphological parameters in IPF patients

**Figure 2:**
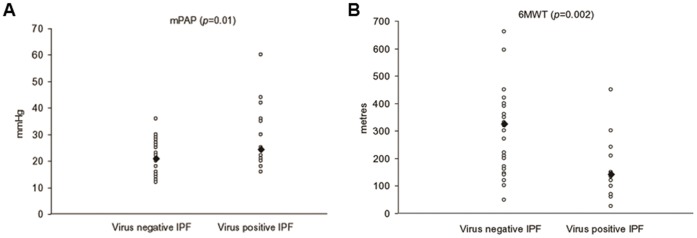
Mean pulmonary arterial pressure (mPAP) values. Significant higher values of mPAP (A) and worse 6MWT (B) in virus positive compared to virus negative cases.

Virus-positive IPF cases showed increased mPAP (28.6±10.9 mmHg vs 21.2±6.0 mmHg, *p = *0.01) and worse performance in the 6MWT (175.2±100 vs 300.5±138.8, *p = *0.002) than virus negative cases (Figure 2 A,B). The statistical value of these associations (mPAP and 6MWT) was still evident when EBV positive IPF patients were compared with both other virus positive and negative IPF cases (p<0.05 for both).

**Figure 3:**
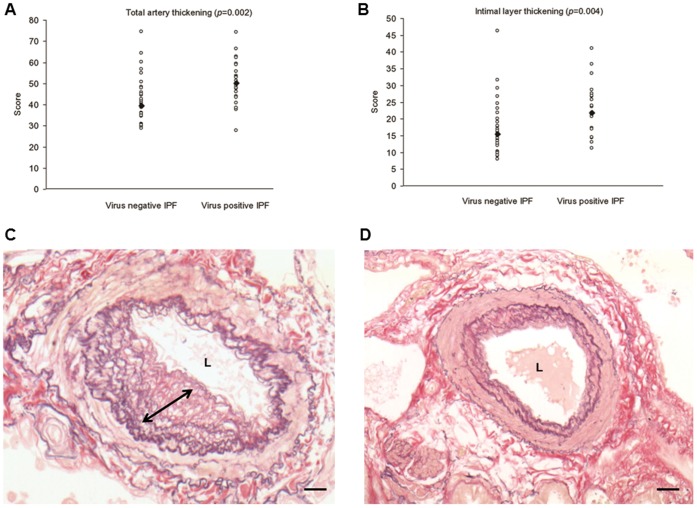
Vascular remodelling in IPF lung tissue. Significantly increased arterial thickening (A) particularly of the intimal layer (B) is seen in virus positive cases. Elastic Van Gieson stained sections showed increased wall thickening (increased elastic and collagen fibers) especially of the intimal layer (arrow) in virus positive (C) compared to virus negative cases (D). Bar scale: 100 µm. L = lumen.

**Figure 4:**
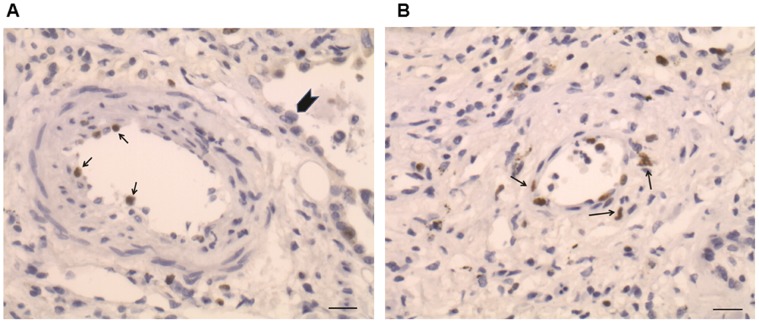
Vessel cell proliferation in IPF lung tissue. Strong Ki-67 immunostaining was observed both in endothelial (A, arrows) and in smooth muscle cells (B, arrows) of remodelled pulmonary arteries adjacent to metaplastic epithelial cells (well seen in A, arrowhead). Bar scale: 10 µm.

**Figure 5:**
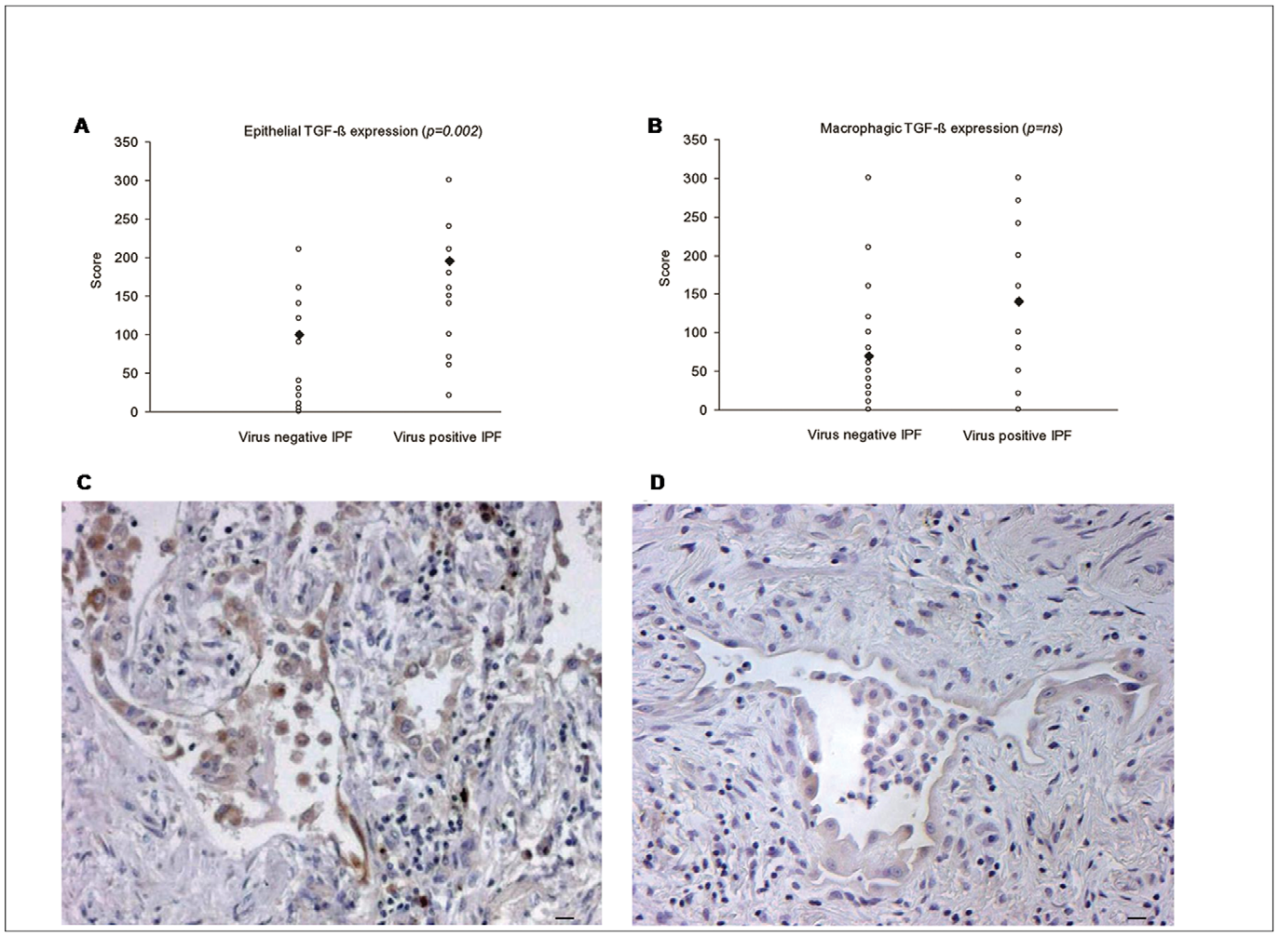
TGF-β expression in IPF lung tissue. A) Significantly increased TGF-β median score values of epithelial cells are seen in virus positive cases. B) TGF-β median score values of macrophages in virus positive and virus negative cases. Stronger and more extensive TGF-β immunostaining well seen in virus positive (C) than virus negative case (D). Bar scale: 5 µm.

Virus positive IPF cases showed a higher total thickening of muscular arteries (50.3%, 43.8–58.8% vs 39.5%, 34.7–45.7%, *p = *0.002) than virus negative cases. The intimal layer was most severely affected (21.8%, 17.2–26.8% vs 15.5%, 12.6–19.1%; *p = *0.004) (Figure 3 A,B,C,D). TUNEL staining was mainly detected in the endothelial cells of the microvasculature (Figure S1) while cell proliferation was frequently seen in remodelled pulmonary arteries. Strong Ki-67 positivity was observed in both endothelial cells (CD31 positive, data not shown) and smooth muscle cells (smooth muscle actin positive, data not shown). Vessel cell proliferation was particularly seen in pulmonary arteries in proximity to metaplastic alveolar epithelial cells or macrophages (Figure 4 A and B). TGF-β scores were higher in virus-positive cases, although this was only statistically significant when epithelial expression was considered (score: 195, 140–210 vs 100, 40–120; *p = *0.002) (Figure 5 A,B,C,D). The statistical value of these parameters (vessel remodelling and TGF-β expression) was still evident when EBV positive IPF patients were compared with both other virus positive and negative IPF cases (p<0.05 for all). A summary of all main clinicopathological features in relation to virus infection among IPF patients is shown in the Table S1.

Viral presence was significantly associated with higher mPAP (*p = *0.03) after adjusting for other related covariates and intermediate factors (i.e.; age, sex, duration of disease, smoking history, and fibrosis extension). Moreover, viral presence was an independent marker of significantly poorer performance in the 6MWT (*p = *0.008) using the GLM adjusting for covariates (i.e. VC, FVC, DLCO and fibrosis extension). When we considered the short-term post-transplant follow-up we found a higher frequency of PGD (50% vs 14%, *p* = 0.02) and, although not statistically significant, an increased rate of acute rejection (50% vs 25%, *p*: ns) in virus positive compared to negative IPF cases. A multivariate analysis identified an increased risk for PGD to be associated with viral infection independently of the major recipient/donor characteristics that are usually considered to influence PGD, including PH (adjusted RR: 5.43, 95% CI: 1.56–7.10, *p* = 0.02) (Table S2).

### MHV-68 Infected CD1 Mice

#### Tissue and vessel remodelling

Analysis of MHV-68 DNA load showed, as previously demonstrated, that viral infection peaked on day 7 post infection [50].

**Figure 6:**
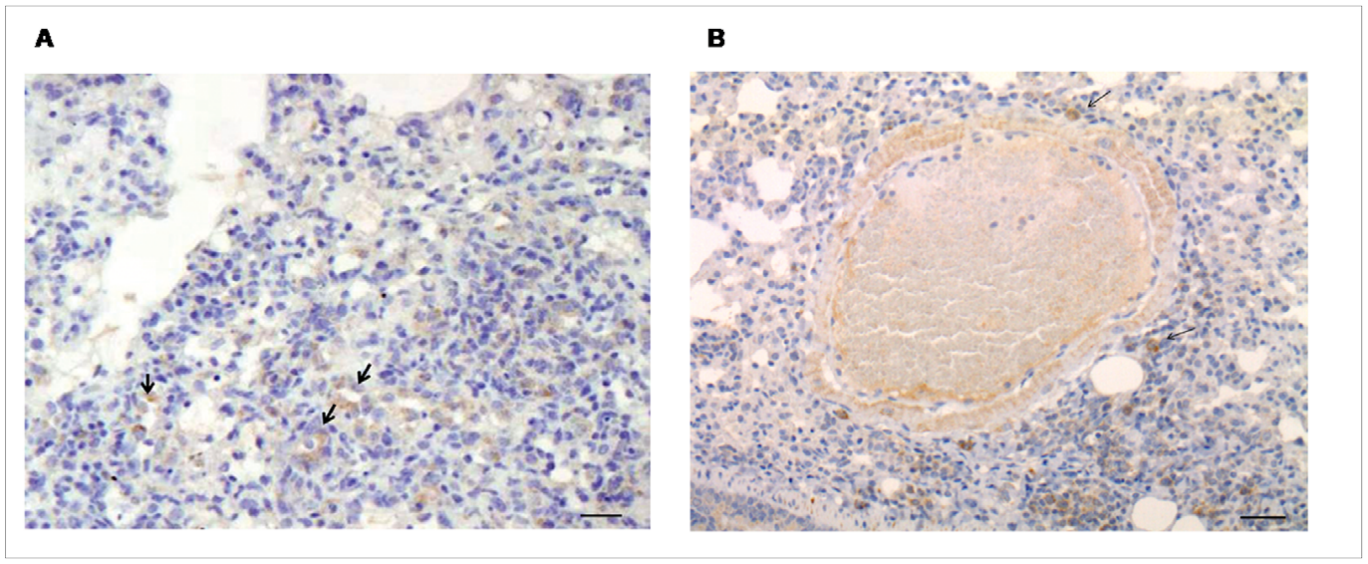
Immunohistochemistry for TGF-β in lung tissue of MHV-68 infected CD1 mice. Numerous metaplastic alveolar epithelial cells (arrows) are marked (A); positive macrophages (arrows) are seen around a remodelled artery (B). Bar scale: 40 µm.

The histological examination of uninfected control animals did not identify any pathological changes. On day 7 post infection, all infected animals exhibited increased, macrophage-dominated interstitial cellularity, mild type II pneumocyte hyperplasia and perivascular inflammatory infiltration. The latter infiltrates were associated with slight patchy collagen deposition. Scattered individual macrophages and metaplastic alveolar epithelial cells were found to express MHV-68 antigen. Viral latency, represented by the expression of viral tRNA, was detected in type II pneumocytes and alveolar macrophages. On day 14 post infection, mild multifocal, almost diffuse fibrosis of alveolar septa was observed with a median of 26% (range: 17–38%). In some animals, this was associated with random patches of collagen deposition. Numerous macrophages were seen within the interstitium and often around arteries. In addition, some type II pneumocytes exhibited TGF-β expression (Figure 6 A, B).

**Figure 7:**
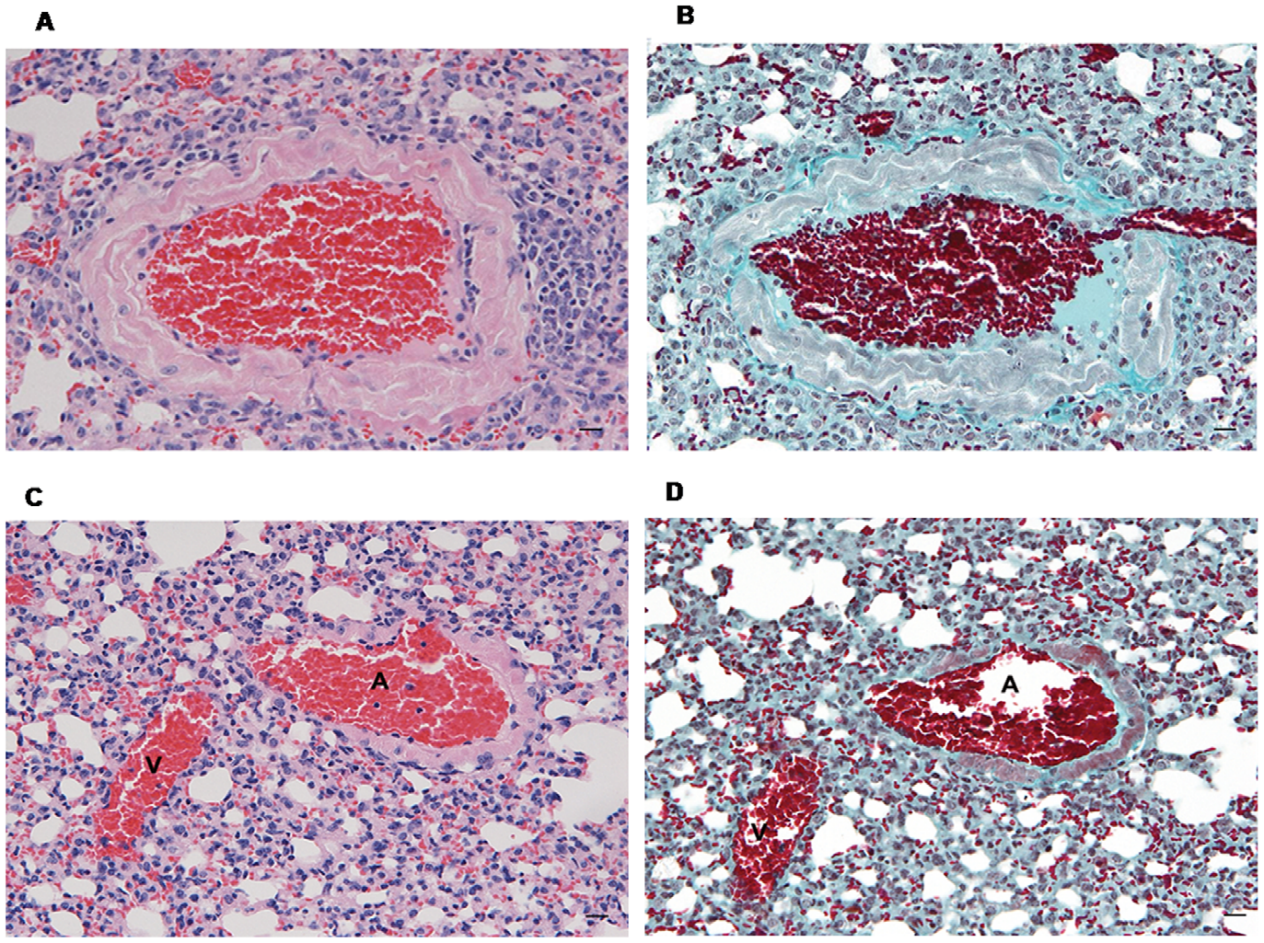
Vascular remodelling in lung tissue of MHV-68 infected CD1mice. Haematoxylin-Eosin (A, C) and Masson’ trichrome (B, D) stained sections: marked arterial thickening is seen in a MHV-68 infected mouse (A and B, scale bars: 20 µm) in comparison to an uninfected mouse (C and D). Bar scale: 20 µm. A: small artery; V: venule.

**Figure 8:**
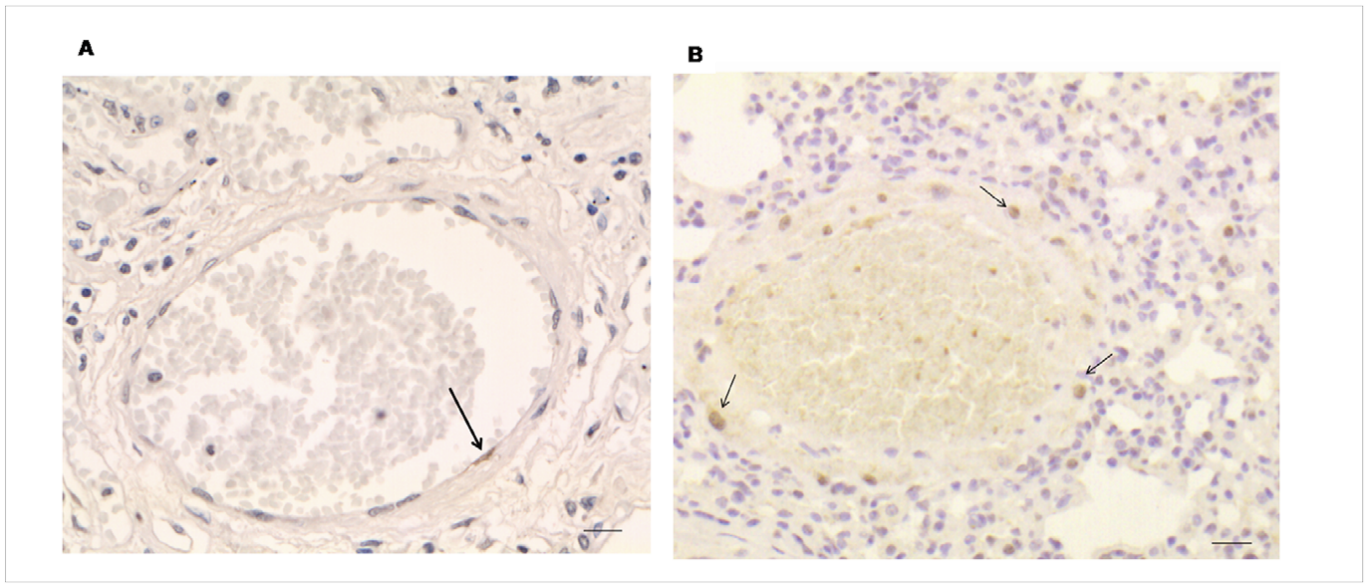
Vessel cell proliferation in lung tissue of MHV-68 infected CD1mice. Strong Ki-67 immunostaining was observed both in endothelial (A, arrow) and smooth muscle (B, arrows) cells adjacent to macrophages. Bar scale: 20 µm.

Arteries were assessed for vessel remodelling and compared for the thickening scores. In uninfected control mice, the average score was 16.5. Seven and 14 days post infection, it was 18 and 24.5 respectively (Figure 7 A,B,C,D). The extent of fibrosis was slightly more marked in mice with vessel wall thickening. On day 23 post infection both the parenchymal and vessel remodelling were less evident. Similar to human cases TUNEL staining was mainly detected in endothelial cells of the microvasculature (Figure S2) while cell proliferation was frequently seen in endothelial cells and smooth muscle cells of pulmonary arteries, in particular in vessels surrounded by macrophages (Figure 8 A, B).

## Discussion

In the present study different respiratory viruses were investigated in lungs from a large cohort of IPF patients and the control group (non IPF DPLDs and normal lungs) displaying the prevalent role of herpes viruses. The work highlights the broad impact of herpesviral infection, particularly EBV, in the disease emphasizing its association with a more severe disease phenotype as IPF with associated higher mPAP and worse 6MWT. A multiple logistic regression analysis demonstrated that herpesviral infection was independently associated with more marked vessel remodelling, higher value of PAP and worse performance in the 6MWT.Neither clinical data (smoking history, age at diagnosis and at transplant, duration of the disease, BMI, lung volumes and hypoxemic respiratory failure) nor morphological changes (fibrosis extension) had more significant impact than viral infection. Of particular note is that the presence of viral infection had also early-term negative influence on the graft function. IPF virus positive recipients showed a higher frequency of PGD, independent of other recipient and donor characteristics. Viral products and/or the activated immune system related to viral infection in native IPF lungs may potentiate the immune response to alloantigens after transplantation, thus resulting in early graft dysfunction. Only one previous study performed on 24 IPF patients reported more rapid disease progression in EBV positive cases. The majority of viral cases died from respiratory failure at a mean of 41 month follow up. In the study PH was not specifically considered [51].

The reported frequency of herpes viruses in IPF lungs ranges from 30 to 100%. These differences may be related to technical sensitivity, disease heterogeneity or selection of patients. The presence of herpes viruses detected in our cases may even be underestimated due the fact that the molecular investigation was performed on tissue samples from end-stage diseases. Viral clearance, after initial injury, could occur in some cases with persistence and progression of lung injury due to activation of an immunological response, as it occurs in other chronic virus-related diseases, e.g. dilated-cardiomyopathy-post-myocarditis [52]. EBV, the most frequent herpes virus detected in IPF, has a well known epitheliotropism other than lymphotropism. Several works have detected EBV in alveolar epithelial cells of IPF lungs, thus confirming the concept that these cells represent the principal viral target in IPF [15], [27], [29], [30], [33], [53]. The role of herpes viruses, important contributing factors for the development of pulmonary fibrosis, has been emphasized in different experimental models and the TGF–β signalling pathway seems to play a crucial role in the profibrogenetic action.

To the best of our knowledge no attempt has been made to specifically investigate the influence of herpes viruses on arterial remodelling and PH in IPF patients.

Several types of viruses, particularly those of the herpes family, have been found to be associated with vessel remodelling, development of atherosclerosis and clinical features of hypertension [54], [55]. Vasculotropism of gamma herpes viruses (such as HHV-8, EHV-5 and MHV68) has been demonstrated in lung parenchyma of patients and animals (horses) with PH even if a causal relationship still remains quite debated [55]–[57]. Up to today, there is little evidence of a “direct” role of viral agents in the pathogenesis of PH. Even when more significant arterial intimal thickness could more convincingly suggest a direct viral endothelial injury, in our work EBV was never detected in endothelial cells of IPF cases. The presence of chemokines and cytokines, viral protein components, and increased expression of growth and transcriptional factors released at the site of infection could contribute to further recruitment of inflammatory cells and proliferation of smooth muscle and endothelial cells. An interesting finding of our study was the most frequent distribution of remodelled vessels with proliferating components in proximity to metaplastic epithelial cells and/or macrophages, both cell types considered as principal targets of EBV. Injured epithelial or endothelial cells involved in tissue and vessel remodelling are considered an important source of growth factors and mediators, among which TGF-β plays a key role.

Although the function of TGF in vascular cell growth in *vivo* has not been well defined, in vitro and experimental studies have demonstrated an important influence of this cytokine in muscle/fibroblast proliferation, endothelial-mesenchymal transition, and extracellular matrix production of intimal and medial layers [58]. In our study significantly higher TGF-β levels detected in our viral IPF cases as well as in MHV-68 infected mice suggest an indirect influence of viral infection on vessel remodelling through this cytokine even if TGF-β expression was not significantly related to arterial thickening. Similar data were found by Farkas L. et al. in a different experimental model of pulmonary fibrosis [13]. The authors detected high levels of active TGF-β in areas with increased fibrogenesis and pulmonary artery remodelling. At day 14, this was significantly associated with pulmonary hypertension.

The demonstration of a direct causal relationship between herpesvirus infection and vessel remodelling/PH in IPF would require longitudinal studies of the same patients, an impossible task with lung tissue but attainable with bronchoalveolar lavage or peripheral blood samples. However this limitation has been partially overcome in the present study using a laboratory MHV-68 infected mouse model. Indeed, in these animals 2 weeks after infection significant arterial remodelling and increased TGF-β expression was seen, as those observed in clinical lung specimens from IPF patients with high mPAP.

### Conclusion

In summary, our results demonstrated for the first time a different phenotype of virus-positive IPF patients. In particular virus-positive IPF cases showed more pronounced vessel remodelling and a higher mPAP and significantly higher PGD after transplantation. While there is large mechanistic evidence of epithelial herpesvirus-associated alveolar injury, the effect of these viruses on the pulmonary vasculature in IPF merits investigation. A deeper knowledge of viral-induced pathways in endothelial cells could give new insights for a targeted therapeutic approach of this important complication in the subgroup of patients (virus positive cases). In this context, the high degree of similarity between MHV-68 infection of CD-1 mice and virus positive IPF indicates that this is an excellent model with which to study pathogenesis and interventions.

## Supporting Information

Figure S1 **Cell apoptosis in IPF lung tissue.** Endothelial cell apoptosis (TUNEL positive) well seen in a capillary surrounded by extensive fibrosis (arrows). Bar scale: 10 µm.
(TIF)

Figure S2 **Cell apoptosis in MHV-68 infected CD1 mice lungs.** Endothelial cell apoptosis (TUNEL positive, arrow) well seen in a capillary of high remodeled area. Note apoptotic body inside a macrophage (arrow head). Bar scale: 10 µm.
(TIF)

Table S1 Virus-positive vs virus-negative IPF (clinical/pathological correlations).
(DOC)

Table S2 Unadjusted relative risks (95% confidence interval) for post-transplant PGD – recipients and donors characteristics used as predictors.
(DOC)
