# Supplementary figures and images for: Herpes Virus Infection Is Associated with Vascular Remodeling and Pulmonary Hypertension in Idiopathic Pulmonary Fibrosis
Source: PLoS One. 2013 Feb 28;8(2):e55715. doi: 10.1371/journal.pone.0055715 (PMC3585298; doi:10.1371/journal.pone.0055715)

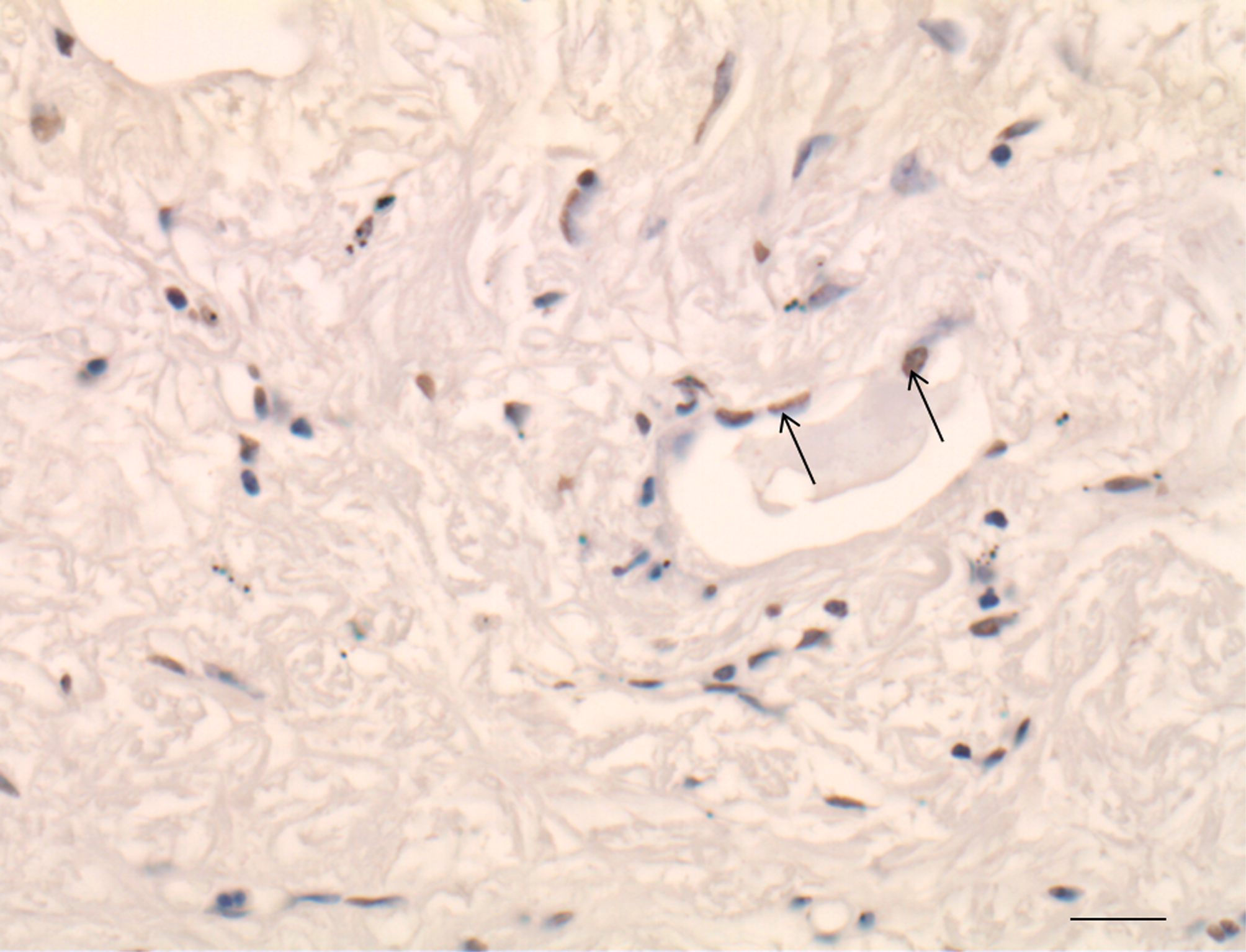

Supplement: Figure S1 — Cell apoptosis in IPF lung tissue. Endothelial cell apoptosis (TUNEL positive) well seen in a capillary surrounded by extensive fibrosis (arrows). Bar scale: 10 µm. (TIF) [file pone.0055715.s001.tif]

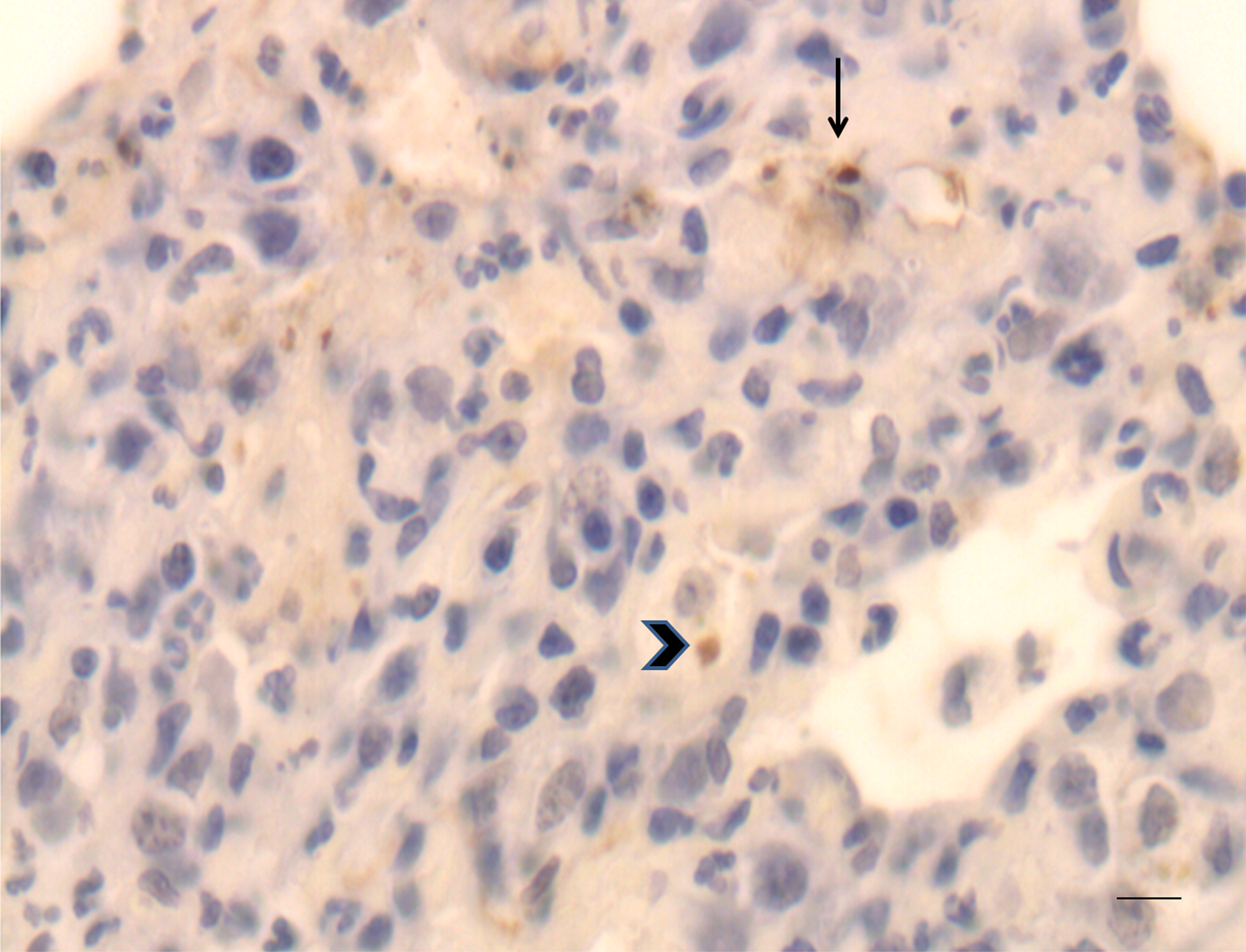

Supplement: Figure S2 — Cell apoptosis in MHV-68 infected CD1 mice lungs. Endothelial cell apoptosis (TUNEL positive, arrow) well seen in a capillary of high remodeled area. Note apoptotic body inside a macrophage (arrow head). Bar scale: 10 µm. (TIF) [file pone.0055715.s002.tif]
