# Supplementary material for: Herpes Virus Infection Is Associated with Vascular Remodeling and Pulmonary Hypertension in Idiopathic Pulmonary Fibrosis
Source: PLoS One. 2013 Feb 28;8(2):e55715. doi: 10.1371/journal.pone.0055715 (PMC3585298; doi:10.1371/journal.pone.0055715)
Supplement: Table S1 — Virus-positive vs virus-negative IPF (clinical/pathological correlations). (DOC) [file pone.0055715.s003.doc]

**Table S1** Virus-positive vs virus-negative IPF (clinical/pathological correlation)

|  |  |  |  |
| --- | --- | --- | --- |
|  | **Virus positive**  **(n=22)** | **Virus negative**  **(n=33)** | ***p values*** |
|  |  |  |  |
| Age at diagnosis (years) | 50.5 (7.8) | 50.7 (8.1) | *ns* |
| Age at transplantation (years) | 54.7 (8.6) | 55.3 (7.6) | *ns* |
| Men | 16 (72.7%) | 23 (69.7%) | *ns* |
| BMI | 28.3 (3.9) | 26.4 (4.1) | *ns* |
| Smoking status |  |  | *ns* |
| never smoked | 6 (27.2%) | 3 (9.0%) | *ns* |
| previous smoker | 16 (72.8%) | 30 (91.0%) | *ns* |
| pack/years | 23.5 (30.2) | 22.1 (19.3) | *ns* |
| Time from symptoms to transplantation (months) | 61.5 (35.0) | 59.3 (32.0) | *ns* |
| Family history of IPF | 0 | 0 | *-* |
| FEV1 | 49.2 (16.3) | 47.1 (16.4) | *ns* |
| FVC | 44.6 (14.5) | 45.8 (14.3) | *ns* |
| VC | 43.8 (14.2) | 43.2 (14.6) | *ns* |
| TLC | 52.7 (15.6) | 53.9 (15.8) | *ns* |
| DLco | 21.3 (12.7) | 28.6 (18.7) | *ns* |
| Mean PAP (mmHg) | 28.6 (10.9) | 21.2 (6.0) | *0.01* |
| Distance of 6-min-walk (m) | 175.2 (100) | 300.5 (138.8) | *0.002* |
| Use of supplemental oxygen | 21 (95.4%) | 29 (87.8%) | *ns* |
| Use of prednisolone or equivalent | 16 (72.7%) | 22 (66.7%) | *ns* |
| Fibrotic extension | 34.8 (8.8) | 38.1 (14.3) | *ns* |
| Median arterial remodelling | 26.3 (24.5-31.3) | 25.3 (20.8-28.0) | *ns* |
| Intimal arterial remodelling | 21.8 (17.2-26.8) | 15.5 (12.6-19.1) | *0.004* |
| Total arterial remodelling | 50.3 (43.8-58.8) | 39.5(34.7-45.7) | *0.002* |
| Epithelial TGF-β expression | 195 (140-210) | 100 (40-120) | *0.002* |
| Macrophagic TGF-β expression | 140 (20-240) | 70 (30-160) | *ns* |

Data are number of patients (%) or mean (SE). IPF: idiopathic pulmonary fibrosis; BMI: body mass index; TGF: transforming growth factor.
