# Supplementary material for: Herpes Virus Infection Is Associated with Vascular Remodeling and Pulmonary Hypertension in Idiopathic Pulmonary Fibrosis
Source: PLoS One. 2013 Feb 28;8(2):e55715. doi: 10.1371/journal.pone.0055715 (PMC3585298; doi:10.1371/journal.pone.0055715)
Supplement: Table S2 — Unadjusted relative risks (95% confidence interval) for post-transplant PGD – recipients and donors characteristics used as predictors. (DOC) [file pone.0055715.s004.doc]

|  | **Variable** | **RR** | **95%CI** | ***p*** |
| --- | --- | --- | --- | --- |
| **Recipient** | Sex | 0.54 | 0.14 – 1.36 | 0.25 |
|  | Age ≥ 50 years | 0.95 | 0.25 – 2.20 | 0.93 |
|  | BMI >30 | 0.98 | 0.26 – 1.73 | 0.96 |
|  | ARI> 50 | 0.61 | 0.15 – 1.62 | 0.40 |
|  | Virus | 3.67 | 1.27 – 6.06 | **0.02** |
|  | PAP ≥ 25mmHg | 2.51 | 0.82 – 3.90 | 0.09 |
|  | Type of transplant (*BLT vs SLT)* | 1.35 | 0.42 – 2.67 | 0.57 |
| **Donor** | Age ≥ 45 years) | 0.69 | 0.08 – 2.17 | 0.66 |
|  | Marginal | 0.44 | 0.11 – 1.25 | 0.15 |
|  | Sex | 0.93 | 0.25 – 2.01 | 0.90 |
|  | Ischemia time (>480 min) | 1.22 | 0.12 – 3.25 | 0.84 |

**Table S2. Unadjusted relative risks (95% confidence interval) for post-transplant PGD – recipients and donors characteristics used as predictors.**

RR: relative risk, CI: confidence interval; BMI: body mass index; ARI: acute rejection index, calculated as the number of acute rejection/total number of transbronchial biopsies; PAP: pulmonary artery pressure; BLT: bilateral lung transplantation; SLT: single lung transplantation.
